# Endothelial cell‐specific progerin expression does not cause cardiovascular alterations and premature death

**DOI:** 10.1111/acel.14389

**Published:** 2024-10-31

**Authors:** Ignacio Benedicto, Magda R. Hamczyk, Rosa M. Nevado, Ana Barettino, Rosa M. Carmona, Carla Espinós‐Estévez, Pilar Gonzalo, Miguel de la Fuente‐Pérez, María J. Andrés‐Manzano, Cristina González‐Gómez, Beatriz Dorado, Vicente Andrés

**Affiliations:** ^1^ Centro de Investigaciones Biológicas Margarita Salas (CIB), Consejo Superior de Investigaciones Científicas (CSIC) Madrid Spain; ^2^ Centro Nacional de Investigaciones Cardiovasculares (CNIC) Madrid Spain; ^3^ CIBER en Enfermedades Cardiovasculares (CIBERCV) Madrid Spain; ^4^ Departamento de Bioquímica y Biología Molecular, Instituto Universitario de Oncología (IUOPA) Universidad de Oviedo Oviedo Spain; ^5^ Aarhus Institute of Advanced Studies (AIAS) Aarhus University Aarhus Denmark

**Keywords:** atherosclerosis, cardiovascular disease, endothelial cells, Hutchinson‐Gilford progeria syndrome, progerin

## Abstract

Hutchinson‐Gilford progeria syndrome (HGPS) is a rare genetic disorder caused by a mutation in the *LMNA* gene that provokes the synthesis of progerin, a mutant version of the nuclear protein lamin A that accelerates aging and precipitates death. The most clinically relevant feature of HGPS is the development of cardiac anomalies and severe vascular alterations, including massive loss of vascular smooth muscle cells, increased fibrosis, and generalized atherosclerosis. However, it is unclear if progerin expression in endothelial cells (ECs) causes the cardiovascular manifestations of HGPS. To tackle this question, we generated atherosclerosis‐free mice (*Lmna* ^*LCS/LCS*^ *Cdh5‐CreERT2*) and atheroprone mice (*Apoe* ^*−/−*^ *Lmna* ^*LCS/LCS*^ *Cdh5‐CreERT2*) with EC‐specific progerin expression. Like progerin‐free controls, *Lmna* ^*LCS/LCS*^ *Cdh5‐CreERT2* mice did not develop heart fibrosis or cardiac electrical and functional alterations, and had normal vascular structure, body weight, and lifespan. Similarly, atheroprone *Apoe* ^*−/−*^ *Lmna* ^*LCS/LCS*^ *Cdh5‐CreERT2* mice showed no alteration in body weight or lifespan versus *Apoe* ^*−/−*^ *Lmna* ^*LCS/LCS*^ controls and did not develop vascular alterations or aggravated atherosclerosis. Our results indicate that progerin expression in ECs is not sufficient to cause the cardiovascular phenotype and premature death associated with progeria.

## Graphical abstract

Hutchinson‐Gilford progeria syndrome (HGPS) is a rare genetic disorder caused by progerin, a mutant protein that is expressed in multiple cell types and accelerates aging, induces cardiovascular disease, and precipitates death. This study shows that mice with progerin expression restricted to endothelial cells do not develop heart fibrosis, cardiac electrical or functional alterations, or aggravated atherosclerosis, and have normal vascular structure, body weight, and lifespan.

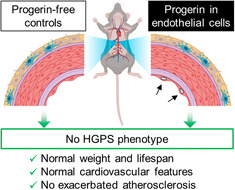

**Abbreviations**
ECs: endothelial cells
HGPS: Hutchinson‐Gilford progeria syndrome
VSMCs: vascular smooth muscle cells

## 1 INTRODUCTION

Hutchinson‐Gilford progeria syndrome (HGPS) is an ultrarare genetic disease affecting 1 in 19 million people that induces accelerated aging and premature death at an average age of 14.5 years (Gordon et al., 1993). In most HGPS patients, progeria is caused by a heterozygous de novo point mutation in the *LMNA* gene (c.1824C > T, p.G608G) that generates an aberrant splicing site which promotes the expression of progerin, a truncated version of the nuclear protein lamin A (De Sandre‐Giovannoli et al., 2003; Eriksson et al., 2003). Progerin is widely expressed in most cell types of HGPS patients and induces multiple molecular, cellular, and functional alterations that ultimately lead to accelerated organismal aging (Benedicto et al., 2021; Hamczyk, del Campo, & Andres, 2018). HGPS patients are born without symptoms and typically begin to develop signs of the disease during the first 2 years of life. These signs include body weight loss, failure to thrive, alopecia, bone and muscle abnormalities, skin alterations, joint contractures, and lipodystrophy, all of which worsen over time. However, the most clinically relevant feature of HGPS is the development of severe cardiovascular alterations, with the main cause of death in HGPS patients being myocardial infarction, heart failure, or stroke (Gordon et al., 1993).

The HGPS cardiovascular phenotype is defined by cardiac repolarization anomalies, left ventricular diastolic dysfunction, heart‐valve disease, vascular stiffening, calcification and fibrosis, loss of vascular smooth muscle cells (VSMCs), and generalized atherosclerosis [reviewed in (Benedicto et al., 2021; Gordon et al., 1993; Hamczyk, del Campo, & Andres, 2018)]. Cardiovascular physiology is intimately influenced by the endothelial cells (ECs) and VSMCs of the vessel wall, and understanding the mechanisms underlying the cardiovascular alterations in HGPS thus requires investigation of the effects of progerin expression in these cell types (McClintock et al., 2006; Olive et al., 2010). Our previous mouse studies showed that *SM22α* promoter‐driven progerin expression in VSMCs of *Apoe*‐deficient mice is sufficient to induce VSMC loss, vascular stiffening, adventitial thickening, and impaired arterial contraction and to aggravate atherosclerosis and provoke premature death (Del Campo et al., 2019, 2020; Hamczyk, Villa‐Bellosta, et al., 2018). ECs, the other main component of the vascular wall, play key roles in healthy and diseased blood vessels, including the maintenance of vascular tone and compliance, the regulation of vascular inflammation, and the development of atherosclerosis (Xu et al., 2021). Here, we used conditional mouse models to test whether EC‐specific progerin expression is sufficient to induce HGPS‐associated cardiovascular pathology and premature death.

## 2 METHODS

### 2.1 Mice

**FIGURE 1:**
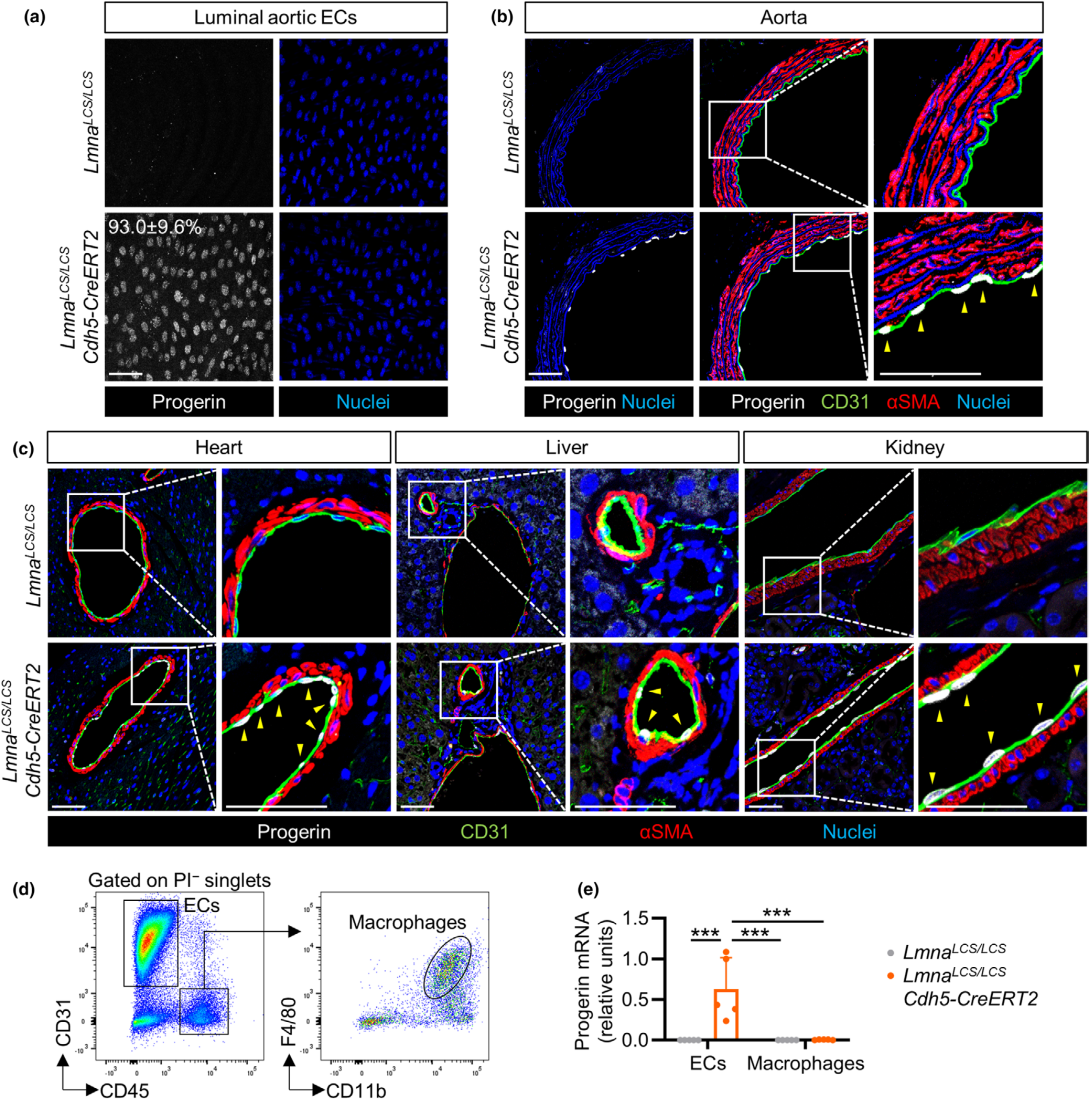
Endothelium‐specific progerin expression in *Lmna* ^*LCS/LCS*^ *Cdh5‐CreERT2* mice. (a) *En face* immunofluorescence staining of thoracic aortas from 22‐month‐old *Lmna* ^*LCS/LCS*^ and *Lmna* ^*LCS/LCS*^ *Cdh5‐CreERT2* mice, showing progerin (white) and nuclei (blue). The percentage of progerin‐positive ECs in *Lmna* ^*LCS/LCS*^ *Cdh5‐CreERT2* mice is indicated (*n* = 4). Luminal aortic ECs from *Lmna* ^*LCS/LCS*^ *Cdh5‐CreERT2* mice were considered progerin‐positive when nuclear progerin expression was above the mean value in *Lmna* ^*LCS/LCS*^ control mice. Bar, 50 μm. (b, c) Immunofluorescence analysis of cross‐sections from thoracic aorta (b), heart, liver, and kidney (c) of 22‐month‐old mice, showing progerin (white), CD31 (green), αSMA (red), and nuclei (blue) (*n* = 4). Bars, 50 μm. Yellow arrowheads indicate examples of progerin‐positive ECs. (d) Cell sorting gating strategy for the isolation of viable cardiac ECs (PI^−^CD31^+^CD45^−^) and macrophages (PI^−^CD31^−^CD45^+^CD11b^+^F4/80^+^). PI, propidium iodide. (e) RT‐qPCR analysis of progerin expression in cardiac ECs and macrophages from 22‐month‐old mice (*n* = 5, two‐way ANOVA plus Tukey's multiple comparisons test). ***p < 0.001.

All experimental mice were on the C57BL/6J genetic background. *Lmna* ^*LCS/LCS*^ *Cdh5‐CreERT2* mice were generated by crossing *Lmna* ^*LCS/LCS*^ (Osorio et al., 2011) and *Tg(Cdh5‐cre/ERT2)1Rha* mice (MGI ID 3848982) (Sörensen et al., 2009). Atherosusceptible *Apoe* ^*−/−*^ *Lmna* ^*LCS/LCS*^ *Cdh5‐CreERT2* mice were generated by crossing *Apoe* ^*−/−*^ *Lmna* ^*LCS/LCS*^ mice (Hamczyk et al., 2018) and *Tg(Cdh5‐cre/ERT2)1Rha* mice. At 1.5 months of age, all mice received daily intraperitoneal injections of 0.2 mg/g tamoxifen (T5648, Sigma) in corn oil (C8267, Sigma) for 3 days. Mice were housed in a specific pathogen‐free facility in individually ventilated cages with a 12 h light/12 h dark cycle at 22 ± 2°C and 50% relative humidity (range 45%–60%). Mice had ad libitum access to water and food (5 K67, LabDiet, D184, SAFE, and Rod18‐A, LASQCdiet). Where indicated, *Apoe* ^*−/−*^ *Lmna* ^*LCS/LCS*^ and *Apoe* ^*−/−*^ *Lmna* ^*LCS/LCS*^ *Cdh5‐CreERT2* mice were placed at 2 months of age on a high‐fat diet containing 10.7% total fat and 0.75% cholesterol (S9167‐E10/E011, Ssniff) and were sacrificed at 4 months of age for post‐mortem analysis. For experiments with atheroresistant *Lmna* ^*LCS/LCS*^ and *Lmna* ^*LCS/LCS*^ *Cdh5‐CreERT2* mice, males and females were used. For experiments with atherosusceptible *Apoe* ^*−/−*^ *Lmna* ^*LCS/LCS*^ and *Apoe* ^*−/−*^ *Lmna* ^*LCS/LCS*^ *Cdh5‐CreERT2* mice, only males were used. All analyses were carried out by operators blinded to genotype. Mice were euthanized by CO_2_ inhalation except for the immunofluorescence experiments shown in Figures 1a–c, 4c, and 5a, where animals were anesthetized and perfused with 4% formaldehyde. All animal procedures followed EU Directive 2010/63/EU and Recommendation 2007/526/EC, enacted in Spain under Real Decreto 53/2013 and 191/2013. Animal protocols were approved by the local ethics committees and the Animal Protection Area of the Comunidad Autónoma de Madrid (PROEX 105.0/22, PROEX76/14, and PROEX149.0/20).

### 2.2 Aorta *en face* immunostaining and quantitative image analysis

*En face* immunofluorescence studies in mouse thoracic aortas were performed as described (Barettino et al., 2022) with a minor modification to the anesthesia procedure for atheroprone *Apoe* ^*−/−*^ *Lmna* ^*LCS/LCS*^ and *Apoe* ^*−/−*^ *Lmna* ^*LCS/LCS*^ *Cdh5‐CreERT2* mice, which were anesthetized by intraperitoneal injection of a pentobarbital (Dolethal, Vetoquinol) and lidocaine (B. Braun Medical S.A.) solution in saline (150 and 1.5 mg/kg, respectively). The aortic luminal face was stained with a rabbit antibody specific to progerin (Rolas et al., 2024) (1:800) (Figure 1a), rabbit anti‐lamin A (which also detects progerin) (1:100, sc‐20680, Santa Cruz) (Figure 5a), and biotinylated mouse anti‐mouse CD45.2 (1:200, 553,771, BD Biosciences). Note that *Lmna* ^*LCS/LCS*^ mice and derivatives do not express lamin A and progerin expression is induced in these models upon Cre‐mediated recombination (Osorio et al., 2011); therefore, the sc‐20680 antibody shows no reactivity with tissues from *Apoe* ^*−/−*^ *Lmna* ^*LCS/LCS*^ controls and detects progerin in ECs from *Apoe* ^*−/−*^ *Lmna* ^*LCS/LCS*^ *Cdh5‐CreERT2* mice (Figure 5a). Bound primary antibodies were detected with goat anti‐rabbit IgG H&L Alexa Fluor 647 (1:400, A21245, Thermo Fisher Scientific) and streptavidin Cyanine Cy3 (1:200, 016–160‐084, Jackson ImmunoResearch), and nuclei were stained with Hoechst 33342 (10 μM, B2261, Sigma). Images were acquired with an LSM 700 confocal microscope equipped with a LD LCI Plan‐Apochromat 25×/0.8 Imm Korr DIC M27 objective (Zeiss). Images of 2–3 25× fields were obtained in three zones along the length of the thoracic aorta and were quantified using ImageJ Fiji software (Schindelin et al., 2012). To quantify progerin expression, nuclei of luminal ECs (60–133 nuclei per field) were identified and selected by Hoechst 33342 staining, and the mean fluorescence intensity of nuclear progerin was quantified from a sum projection of z‐slices. Luminal aortic ECs from *Lmna* ^*LCS/LCS*^ *Cdh5‐CreERT2* and *Apoe* ^*−/−*^ *Lmna* ^*LCS/LCS*^ *Cdh5‐CreERT2* mice were considered progerin‐positive when the mean nuclear progerin signal was above that in their respective controls. Intimal leukocyte accumulation was estimated by counting CD45^+^ leukocytes in randomly selected fields in each aorta zone. The ImageJ Fiji software was used to analyze the area, perimeter, and shape descriptors of nuclei from luminal ECs in wholemount preparations of thoracic aorta stained with Hoechst 33342.

### 2.3 Immunostaining and confocal imaging of tissue sections

*Lmna* ^*LCS/LCS*^ and *Lmna* ^*LCS/LCS*^ *Cdh5‐CreERT2* mice were anesthetized by intraperitoneal injection of ketamine (225 mg/kg, Ketamidor, Richter Pharma) and xylazine (15 mg/kg, Nerfasin, Fatro). Tissues were fixed with 4% formaldehyde in PBS first for 5 min by perfusion through the left ventricle and then ex vivo at 4°C for 24 h. *Apoe* ^*−/−*^ *Lmna* ^*LCS/LCS*^ and *Apoe* ^*−/−*^ *Lmna* ^*LCS/LCS*^ *Cdh5‐CreERT2* mice were euthanized by CO_2_ inhalation, and aortas were directly fixed with 4% formaldehyde in PBS. Fixed tissues were dehydrated to xylene, embedded in paraffin, and cut into 4–5‐μm sections with a microtome (RM2245, Leica). Processing of heart, liver, and kidney sections included a 1 h incubation at room temperature (RT) in 100 mM glycine. Antigens were retrieved with 10 mM sodium citrate buffer (pH 6), and samples were blocked for 1 h at RT with 0.3% Triton X‐100, 5% BSA, and 5% normal goat serum in PBS. Antibodies were diluted in PBS supplemented with 0.3% Triton X‐100, 5% BSA, and 2.5% normal goat serum. Samples were incubated overnight at 4°C with antibodies targeting progerin (Rolas et al., 2024) (1:800) (Figure 1b, c), lamin A/progerin (1:100, sc‐20680, Santa Cruz) (Figure 5b), and CD31 (1:50, DIA‐310, Dianova), followed by a 2 h incubation at RT with goat anti‐rabbit IgG Affinipure Fab Fragment Alexa Fluor 647 (1:500, 111–607‐008, Jackson ImmunoResearch), goat anti‐rabbit Alexa Fluor 647 (1:500, A‐21245, Invitrogen), goat anti‐Rat IgG (H + L) Alexa Fluor 488 (1:500, A11006, Thermo Fisher Scientific), and anti–smooth muscle α‐actin‐Cy3 (1:200, C6198, Sigma). After incubation for 30 min at RT with DAPI (1:5000, D23842, Invitrogen), sections were mounted in Fluoromount G imaging medium (00–4958‐02, Thermo Fisher Scientific). Images were acquired with an LSM 700 confocal microscope equipped with a LD LCI Plan‐Apochromat 25×/0.8 Imm Korr DIC M27 objective (Zeiss).

### 2.4 Quantification of progerin mRNA expression in heart ECs and macrophages

Mice were euthanized and gently perfused through the left ventricle with 10 mL cold PBS. Heart apexes were minced and digested at 37°C for 15 min in serum‐free DMEM supplemented with digestion buffer [4× stock: 25 mg/mL collagenase A (Roche), 25 mg/mL dispase II (Roche), 250 μg/mL DNase (Roche), 140 mM NaCl, 5 mM KCl, 2.5 mM phosphate buffer, 10 mM HEPES, 2 mM CaCl_2_, and 1.3 mM MgCl_2_], with thorough pipetting of the samples after 10 min and at the end of the digestion procedure. Digested tissue was filtered through a 70‐μm cell strainer, and dissociated cells were pelleted at 400 × g for 5 min at 4°C. Cells were resuspended in sorting buffer (PBS supplemented with 1% FBS, 2 mM EDTA, 5 mM glucose, and 10 mM HEPES), including an anti‐CD16/CD32 antibody to block Fc receptors (1:200, 101302, Biolegend), and then stained for 30 min on ice with anti‐CD31 Alexa Fluor 647 (1:200, 102416, Biolegend), anti‐CD45 Pacific Blue (1:200, 103126, Biolegend), anti‐CD11b FITC (1:200, AGEL0306, AssayGenie), and anti‐F4/80 PE/Cy7 (1:100, 123114, Biolegend). Cells were then washed with 2 mL cold sorting buffer, pelleted at 400 × g for 5 min at 4°C, resuspended in cold sorting buffer, filtered through a 70‐μm cell strainer, and stained with 1 μg/mL propidium iodide (P4864, Sigma). Viable cardiac ECs (PI^−^CD31^+^CD45^−^) and macrophages (PI^−^CD31^−^CD45^+^CD11b^+^F4/80^+^) were isolated with a FACSAria Cell Sorter (BD Biosciences) and directly lysed upon collection in 750 μL TRI Reagent Solution (AM9738, Invitrogen). RNA was extracted following the manufacturer's instructions until phase separation, when the aqueous phases were diluted 1:1 with 70% ethanol and loaded into RNeasy Mini Kit columns (74106, Qiagen) to continue with the RNeasy Mini Kit protocol. cDNA was synthesized with the High‐Capacity cDNA Reverse Transcription Kit (4368814, Applied Biosystems). Progerin mRNA was quantified by real‐time qPCR in a Bio Rad‐CFX384 Real‐Time PCR System using PowerSYBR Green PCR Master Mix (436659, Thermo Fisher Scientific) and the following primers: progerin, 5′‐tggagcgggagcccagagct‐3′ (boundary between aberrantly spliced *Lmna* exon 11 and exon 12) and 5′‐ttcaggcctgctctcctaag‐3′ (*Lmna* exon 12); *Gapdh* (for normalization by the 2^−^^ΔΔCt^ method), 5′‐aaatggtgaaggtcggtgtg‐3′ and 5′‐gagtggagtcatactggaac‐3′. All qPCR reactions were performed in technical duplicates.

### 2.5 Histology and atherosclerosis studies

Mice were euthanized by CO_2_ inhalation and perfused through the left ventricle with 10 mL PBS. Tissue samples were extracted and fixed in 4% formaldehyde in PBS at 4°C for at least 48 h. For histology studies, hearts and aortas were dehydrated to xylene, embedded in paraffin, and cut into 4–5‐μm sections with a microtome (RM2245, Leica). Collagen deposition in the heart was quantified by Sirius red staining (365548, Sigma), and images were analyzed with ImageJ. Perivascular fibrosis was calculated as μm^2^/vessel by quantifying the mean Sirius red‐positive area surrounding cardiac vessels with a maximum diameter >70 μm (6–15 vessels per heart). Interstitial fibrosis was measured as the mean Sirius red‐positive area in each heart section excluding Sirius red‐positive perivascular and pericardial areas, expressed as a percentage. VSMC number and collagen content in the medial layer of the aortic arch were quantified in sections stained with hematoxylin–eosin or Masson's trichrome, respectively (Hamczyk, Villa‐Bellosta, et al., 2018). Aortic atherosclerosis burden was quantified *en face* after staining the aortic arch and thoracic aorta with 0.2% oil red O (O0625, Sigma) (Hamczyk, Villa‐Bellosta, et al., 2018). The atherosclerotic lesion area at the aortic root was quantified from Van Gieson‐stained sections from three zones: at the beginning, in the middle, and at the end of the aortic valve (Hamczyk, Villa‐Bellosta, et al., 2018).

### 2.6 Hematology and serum lipid profile analysis

Animals were sacrificed by CO_2_ inhalation. Blood was collected from *Lmna* ^*LCS/LCS*^ and *Lmna* ^*LCS/LCS*^ *Cdh5‐CreERT2* mice by cardiac puncture. Blood from fat‐fed *Apoe* ^*−/−*^ *Lmna* ^*LCS/LCS*^ and *Apoe* ^*−/−*^ *Lmna* ^*LCS/LCS*^ *Cdh5‐CreERT2* mice was withdrawn from the inferior vena cava after overnight fasting. For hematological tests, blood was collected in Microvette 100 EDTA tubes (Sarstedt) and analyzed with a PENTRA 80 hematology analyzer (Horiba). For serum lipid profile analysis, blood was collected in plastic tubes, allowed to clot at RT for 30 min, and centrifuged at 1,900 × *g* and 4°C for 10 min. Serum was centrifuged at 4°C for 10 min at 16,100 × *g*, and then analyzed with a Dimension RxL Max Integrated Chemistry System (Siemens Healthineers). All analyses were carried out in a blinded manner by specialized staff at the CNIC Animal Facility.

### 2.7 Electrocardiography and echography studies

Electrocardiography and echography assays were carried out as described (Fanjul et al., 2020).

### 2.8 Statistical analysis

**FIGURE 2:**
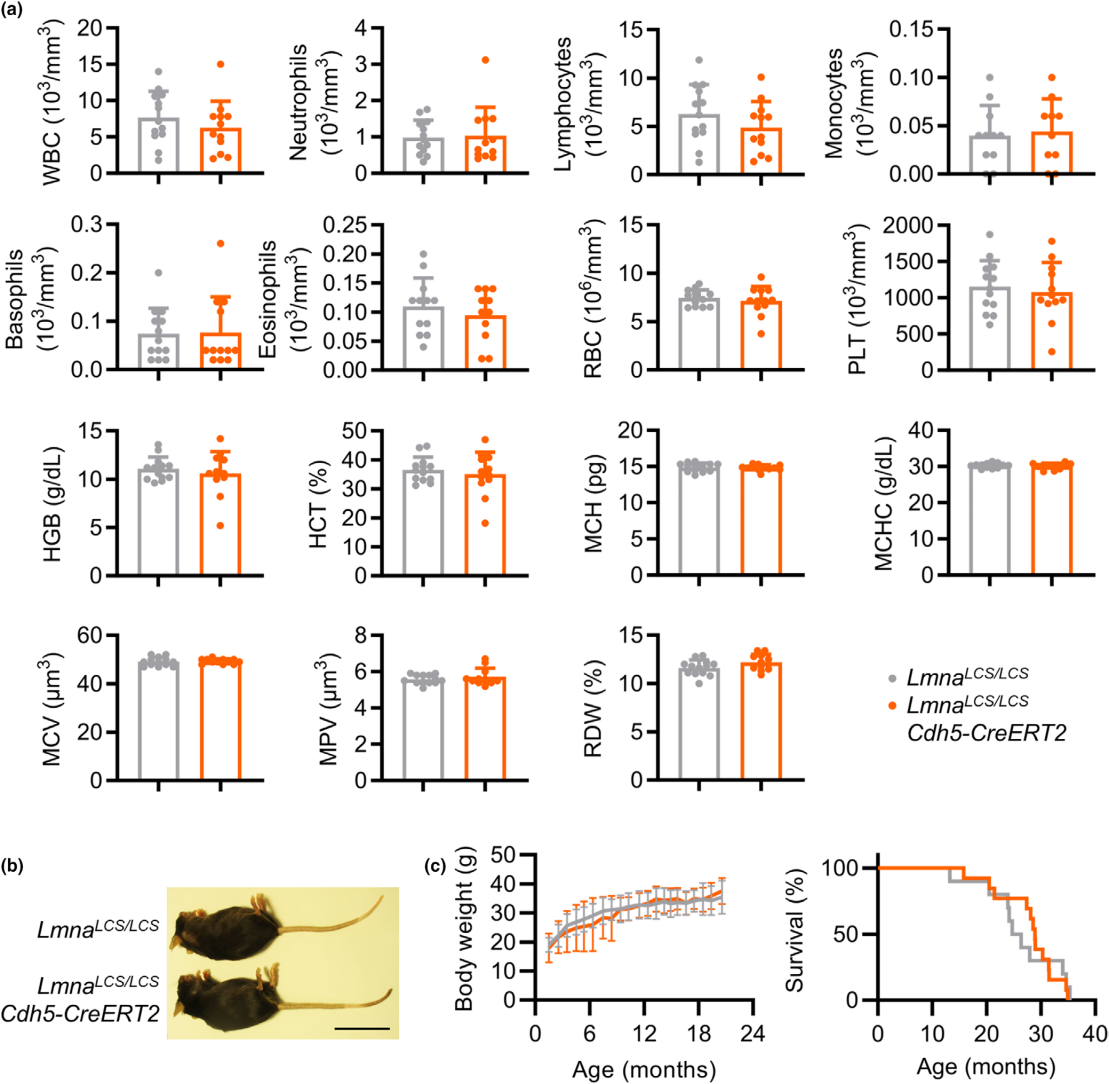
Endothelium‐specific progerin expression in mice does not alter the hematological profile or reduce body weight or lifespan. (a) Hematological profile of 22‐month‐old *Lmna* ^*LCS/LCS*^ and *Lmna* ^*LCS/LCS*^ *Cdh5‐CreERT2* mice (*n* = 12–13, two‐tailed unpaired Student *t*‐test for all parameters except neutrophils, eosinophils, basophils, and MPV, which were analyzed by two‐tailed Mann–Whitney *U*‐test). (b) Representative image of 22‐month‐old female mice. Bar, 5 cm. (c) Body weight (*n* = 8–10, mixed effects model with the Geisser–Greenhouse correction) and Kaplan–Meier survival curves (*n* = 10–13, Mantel–Cox test). WBC, white blood cells; RBC, red blood cells; PLT, platelets; HGB, hemoglobin; HCT, hematocrit; MCV, mean corpuscle volume; MPV, mean platelet volume; MCH, mean corpuscular hemoglobin; MCHC, mean corpuscular hemoglobin concentration; RDW, red cell distribution width.

All data are presented as mean + standard deviation except in Figures 2c and 5d, where the data are presented as mean ± standard deviation. Body weight and Kaplan–Meier survival curves were analyzed using a mixed effects model with the Geisser–Greenhouse correction and a Mantel–Cox test, respectively. For normally distributed data with two groups, we used the two‐tailed unpaired Student *t*‐test, applying Welch's correction if the groups had unequal variances. For non‐normally distributed data with two groups, we used the two‐tailed Mann–Whitney *U*‐test. For data with two independent variables (Figure 1e), we used two‐way ANOVA plus Tukey's multiple comparisons test. Outliers identified using the ROUT test (*Q* = 1%) were excluded from the analysis. Differences were considered significant at *p* < 0.05. Statistical analysis was carried out with Prism 9 (GraphPad).

## 3 RESULTS

### 3.1 Mice with EC‐specific progerin expression have normal body weight and lifespan

To assess whether EC‐specific progerin expression in mice is sufficient to induce HGPS‐associated cardiovascular symptoms, we generated *Lmna* ^*LCS/LCS*^ *Cdh5‐CreERT2* mice by crossing *Lmna* ^*LCS/LCS*^ mice, which enable Cre‐dependent progerin expression (Osorio et al., 2011), with *Cdh5‐CreERT2* mice, in which tamoxifen treatment activates Cre recombinase specifically in ECs (Sörensen et al., 2009). *Lmna* ^*LCS/LCS*^ *Cdh5‐CreERT2* mice and *Lmna* ^*LCS/LCS*^ controls were treated with tamoxifen at 1.5 months of age, and progerin expression was analyzed when the mice were 22 months old. Wholemount immunofluorescence analysis of thoracic aorta of *Lmna* ^*LCS/LCS*^ *Cdh5‐CreERT2* mice showed efficient progerin expression in luminal aortic ECs (93.0 ± 9.6% positive cells) (Figure 1a), which did not exhibit detectable alterations in nuclear area, perimeter, or shape compared with *Lmna* ^*LCS/LCS*^ controls (Figure S1). Progerin expression in ECs was confirmed by immunostaining sections of aorta, heart, liver, and kidney, all of which showed no detectable progerin protein expression in non‐endothelial cell types (Figure 1b,c). To confirm the endothelial specificity of progerin expression in *Lmna* ^*LCS/LCS*^ *Cdh5‐CreERT2* mice, we isolated cardiac ECs (CD31^+^CD45^−^) and macrophages (CD31^−^CD45^+^CD11b^+^F4/80^+^) from *Lmna* ^*LCS/LCS*^ and *Lmna* ^*LCS/LCS*^ *Cdh5‐CreERT2* mice by cell sorting (Figure 1d) and analyzed progerin expression by RT‐qPCR. Progerin mRNA was expressed in cardiac ECs from *Lmna* ^*LCS/LCS*^ *Cdh5‐CreERT2* mice but not in those from *Lmna* ^*LCS/LCS*^ controls and was barely detectable in heart macrophages of either genotype (Figure 1e). *Lmna* ^*LCS/LCS*^ *Cdh5‐CreERT2* mice showed no hematological alterations (Figure 2a) or overt signs of premature aging (Figure 2b), and their body weight and lifespan were indistinguishable from control *Lmna* ^*LCS/LCS*^ mice (Figure 2c).

### 3.2 EC‐specific progerin expression in mice does not induce heart fibrosis or alterations in cardiac electrical activity and function

**FIGURE 3:**
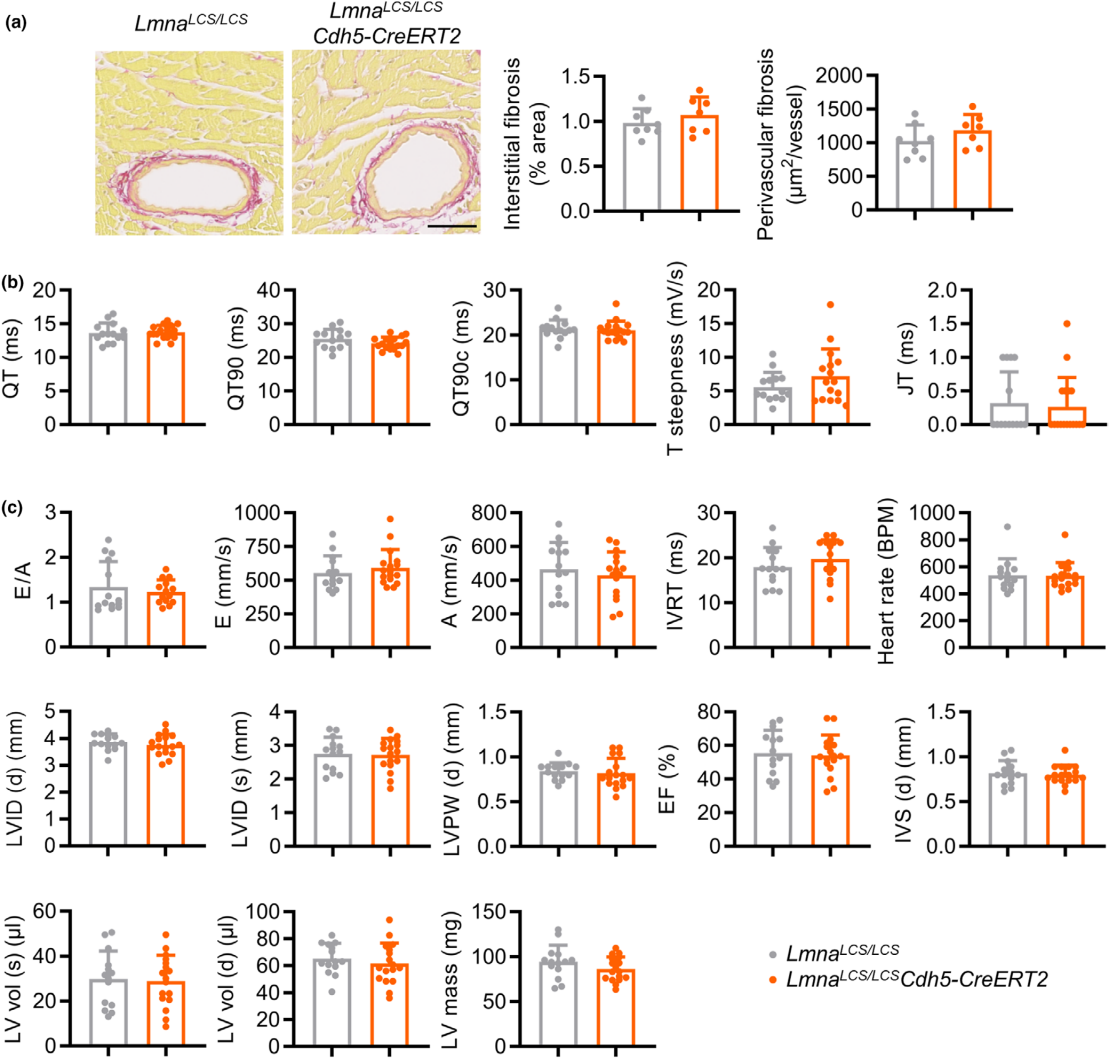
Endothelium‐specific progerin expression in mice induces neither heart fibrosis nor alterations in cardiac electrical activity and function. (a) Collagen content in hearts from 22‐month‐old *Lmna* ^*LCS/LCS*^ and *Lmna* ^*LCS/LCS*^ *Cdh5‐CreERT2* mice, measured by Sirius red staining (*n* = 7–8, two‐tailed unpaired Student *t*‐test). Bar, 50 μm. (b, c) Electrocardiography (b) and echocardiography (c) studies in 18‐month‐old mice [*n* = 14–17, analyzed by two‐tailed unpaired Student *t*‐test [QT, QT90, E, A, IVRT, LVID (d), LVID (s), LVPW (d), EF, IVS (d), LV mass, LV vol (d), and LV vol (s)] or two‐tailed Mann–Whitney *U*‐test (QT90c, T steepness, JT, E/A, and heart rate]. A, mitral valve A velocity; E, mitral valve E velocity; E/A, mitral valve E to A ratio; IVRT, isovolumic relaxation time; LV, left ventricle; IVS (d), inter ventricular septum (diastole); LVID (d), LV internal diameter (diastole); LVID (s), LV internal diameter (systole); LVPW (d), LV posterior wall (diastole); EF, LV ejection fraction; LV vol (d), LV volume (diastole); LV vol (s), LV volume (systole); BPM, beats per minute.

HGPS patients and animal models with ubiquitous progerin expression show extensive cardiac alterations, including heart fibrosis and impaired cardiac electrical activity and function [reviewed in (Benedicto et al., 2021; Gordon et al., 1993; Hamczyk, del Campo, & Andres, 2018)]. We therefore sought to determine whether EC‐specific progerin expression was sufficient to induce HGPS‐associated cardiac pathology. *Lmna* ^*LCS/LCS*^ *Cdh5‐CreERT2* mice did not develop interstitial or perivascular cardiac fibrosis, as shown by Sirius red staining of heart sections from 22‐month‐old mice (Figure 3a). Moreover, electrocardiography studies revealed no differences in cardiac electrical activity between *Lmna* ^*LCS/LCS*^ *Cdh5‐CreERT2* mice and *Lmna* ^*LCS/LCS*^ controls (Figure 3b), and echography assessment showed similar cardiac function in both genotypes (Figure 3c).

### 3.3 EC‐specific progerin expression does not induce VSMC loss, medial fibrosis, or intimal leukocyte accumulation in mouse aorta

**FIGURE 4:**
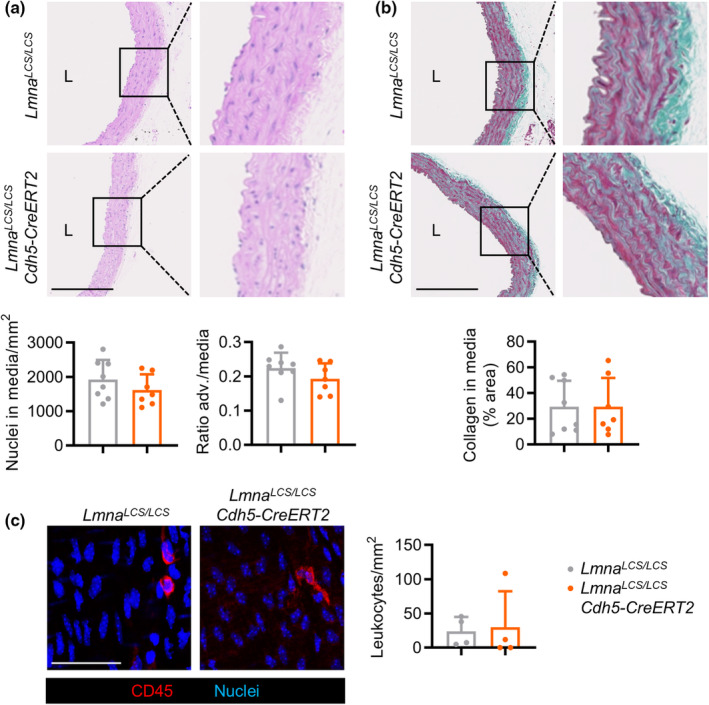
Endothelium‐specific progerin expression in mice does not induce medial vascular smooth muscle cell loss and fibrosis or intimal leukocyte accumulation in the aorta. (a, b) Hematoxylin–eosin (a) and Masson's trichrome (b) staining of aortic arch sections from 22‐month‐old mice to quantify the number of nuclei in the media, the adventitia (adv.)/media thickness ratio, and collagen accumulation in the media (*n* = 7–8, two‐tailed unpaired Student *t*‐test). Bars, 250 μm. (c) *En face* immunostaining of the luminal surface of thoracic aortas, showing CD45^+^ leukocytes (red) and nuclei (blue) (*n* = 4, two‐tailed Mann–Whitney *U*‐test). Bars, 50 μm. L, lumen.

Vascular alterations are major hallmarks of HGPS pathology in animal models and patients and include loss of arterial VSMCs, adventitial thickening, and excessive collagen deposition in the vessel wall [reviewed in (Benedicto et al., 2021; Gordon et al., 1993; Hamczyk, del Campo, & Andres, 2018)]. Moreover, mice with ubiquitous and *SM22α* promoter‐driven progerin expression show intimal leukocyte accumulation (Benedicto et al., 2024; Hamczyk et al., 2024), a marker of vascular inflammation. We therefore studied whether EC‐specific progerin expression could induce these HGPS‐associated vascular alterations. Histological analysis of aortic arch cross‐sections from 22‐month‐old *Lmna* ^*LCS/LCS*^ *Cdh5‐CreERT2* mice revealed no VSMC loss, adventitial thickening, or collagen accumulation in the medial layer in comparison with age‐matched *Lmna* ^*LCS/LCS*^ controls (Figure 4a, b). Likewise, *en face* immunostaining of the thoracic aorta showed no between‐genotype differences in the accumulation of leukocytes in the intimal layer (Figure 4c).

### 3.4 EC‐specific progerin expression does not aggravate atherosclerosis in atheroprone mice

**FIGURE 5:**
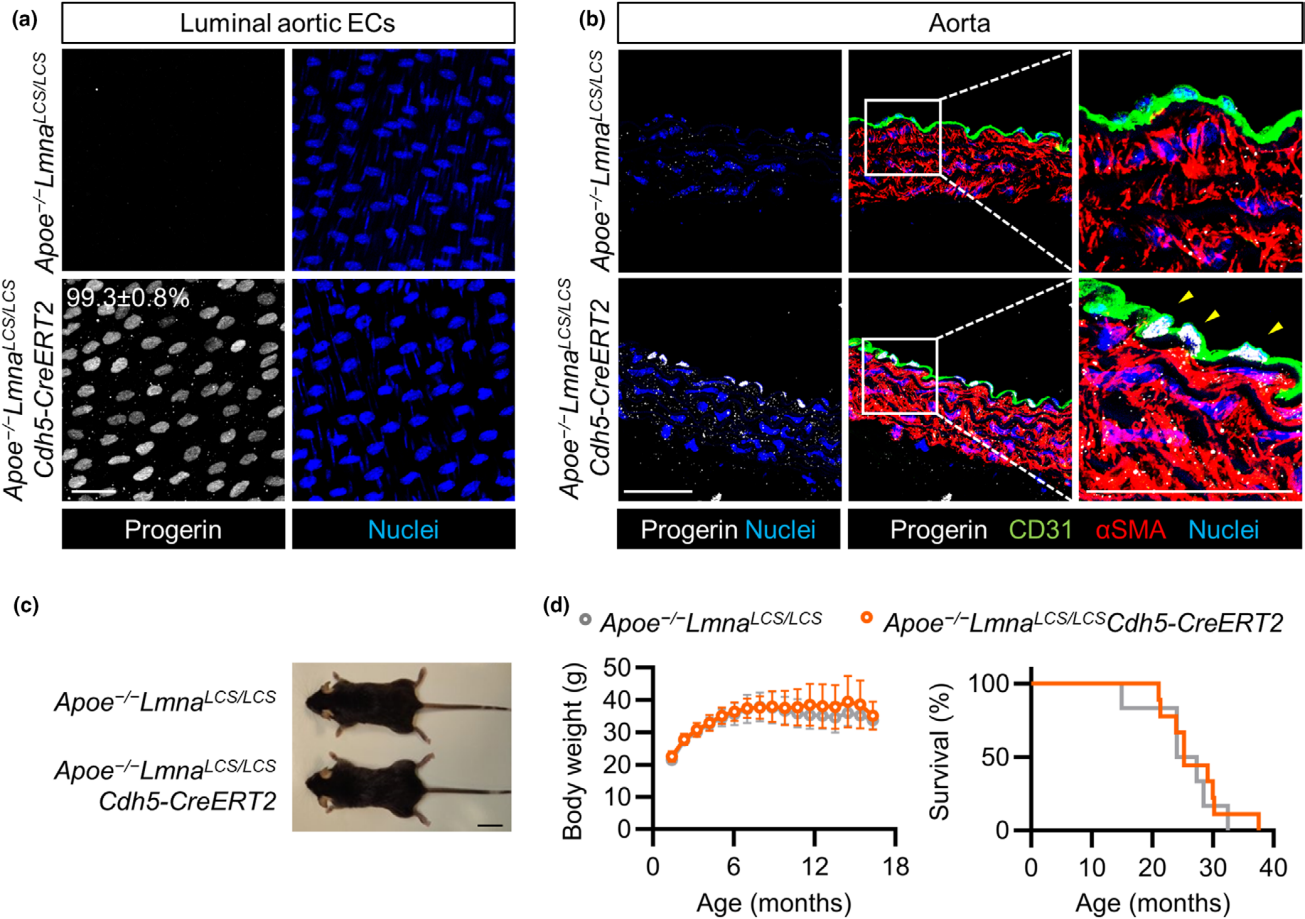
Normal body weight and lifespan in atheroprone mice with endothelium‐specific progerin expression. (a) Progerin expression in *Apoe* ^*−/−*^ *Lmna* ^*LCS/LCS*^ *Cdh5‐CreERT2* mice and *Apoe* ^*−/−*^ *Lmna* ^*LCS/LCS*^ controls fed normal chow was analyzed by *en face* immunofluorescence of thoracic aortas from 4‐month‐old mice (white, progerin; blue, nuclei). The percentage of progerin‐positive ECs in *Apoe* ^*−/−*^ *Lmna* ^*LCS/LCS*^ *Cdh5‐CreERT2* mice is indicated (*n* = 7–8). Luminal aortic ECs in *Apoe* ^*−/−*^ *Lmna* ^*LCS/LCS*^ *Cdh5‐CreERT2* mice were considered progerin‐positive when nuclear progerin expression was above the mean value in *Apoe* ^*−/−*^ *Lmna* ^*LCS/LCS*^ control mice. Bar, 25 μm. (b) Immunofluorescence assays in thoracic aorta sections, showing progerin (white), CD31 (green), αSMA (red), and nuclei (blue) (*n* = 5–6). Bar, 50 μm. Yellow arrowheads indicate examples of progerin‐positive ECs. (c) Representative image of 4‐month‐old male mice. Bar, 2 cm. (d) Body weight (*n* = 6–9, mixed effects model with the Geisser–Greenhouse correction) and Kaplan–Meier survival curves (*n* = 6–9, Mantel–Cox test).

Atherosclerosis is one of the most important clinical features of HGPS, with most patients dying from atherosclerosis complications (Gordon et al., 1993). Progeroid mice with intact *Apoe* and *Ldlr* genes do not develop atherosclerosis, probably due to the intrinsically low levels of pro‐atherogenic lipoproteins in mouse blood (Hamczyk et al., 2018). To circumvent this issue, we previously generated apolipoprotein E–deficient mice with ubiquitous progerin expression (*Apoe* ^*−/−*^ *Lmna* ^*G609G/G609G*^), which developed excessive high‐fat diet‐induced atherosclerosis compared with progerin‐free *Apoe* ^*−/−*^ controls (Hamczyk et al., 2018). To test whether EC‐specific progerin expression aggravated atherosclerosis in atheroprone mice, we generated *Apoe* ^*−/−*^ *Lmna* ^*LCS/LCS*^ *Cdh5‐CreERT2* mice. *Apoe* ^*−/−*^ *Lmna* ^*LCS/LCS*^ *Cdh5‐CreERT2* mice and *Apoe* ^*−/−*^ *Lmna* ^*LCS/LCS*^ controls were injected with tamoxifen at 1.5 months of age, and progerin expression was analyzed in aortas from 4‐month‐old mice. Thoracic aortas from *Apoe* ^*−/−*^ *Lmna* ^*LCS/LCS*^ *Cdh5‐CreERT2* mice showed efficient progerin expression in luminal ECs (99.3 ± 0.8% progerin‐positive cells) (Figure 5a) and undetectable expression in non‐endothelial cell types (Figure 5b). Like atherosclerosis‐free *Lmna* ^*LCS/LCS*^ *Cdh5‐CreERT2* mice (Figures 2b,c and 4c), atherosusceptible *Apoe* ^*−/−*^ *Lmna* ^*LCS/LCS*^ *Cdh5‐CreERT2* mice did not develop signs of accelerated aging (Figure 5c), and their body weight and lifespan were the same as those of *Apoe* ^*−/−*^ *Lmna* ^*LCS/LCS*^ controls (Figure 5d).

**FIGURE 6:**
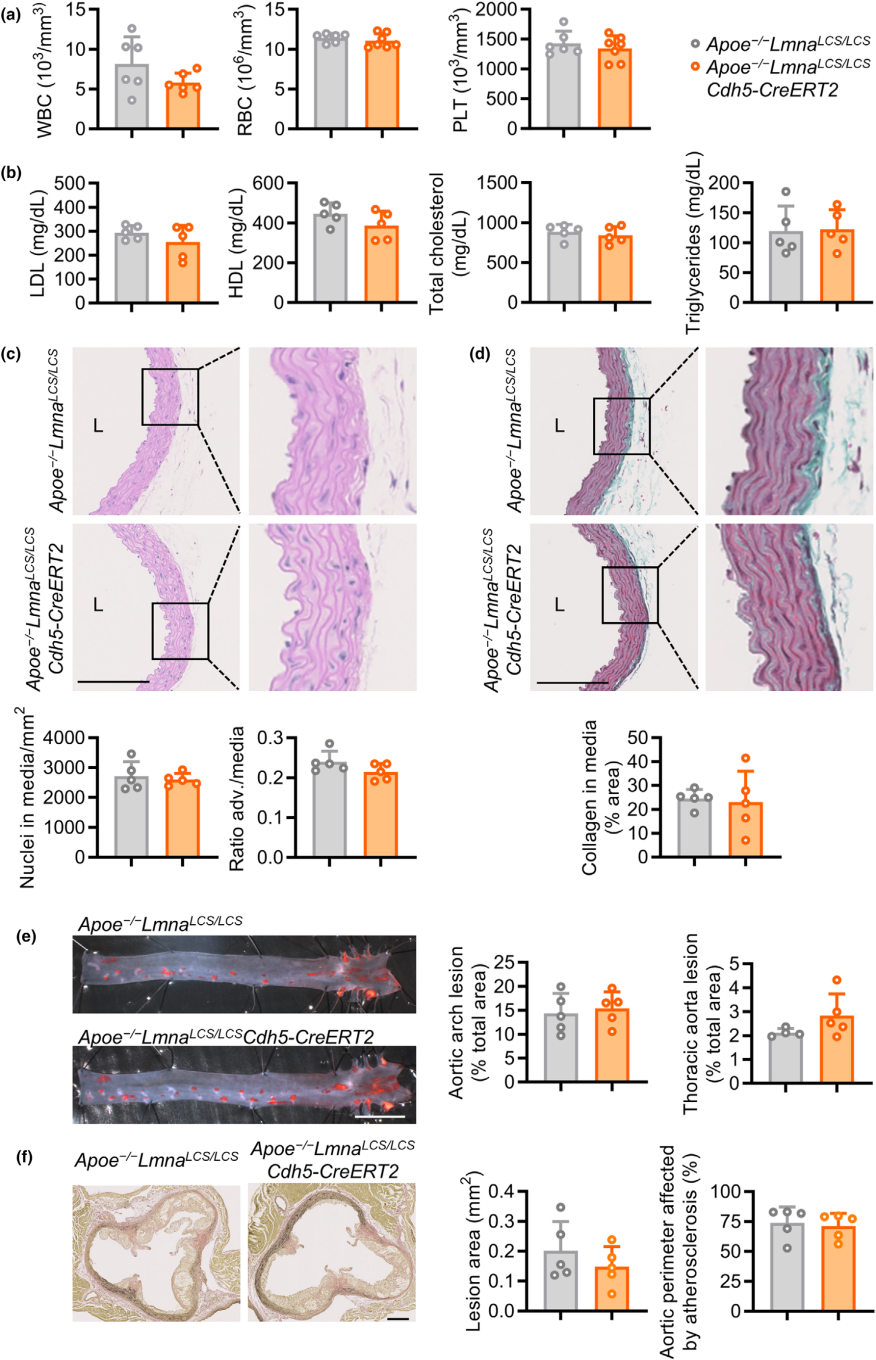
Endothelium‐specific progerin expression does not induce vascular smooth muscle cell loss, medial fibrosis, or aggravated atherosclerosis in atheroprone mice. *Apoe* ^*−/−*^ *Lmna* ^*LCS/LCS*^ and *Apoe* ^*−/−*^ *Lmna* ^*LCS/LCS*^ *Cdh5‐CreERT2* mice were fed a high‐fat diet starting at 2 months of age and sacrificed for analysis at 4 months of age. (a) Hematological profile (*n* = 6–7, two‐tailed unpaired Student *t*‐test for RBC and PLT, and two‐tailed Mann–Whitney *U*‐test for WBC). (b) Serum lipid profile (*n* = 5, two‐tailed unpaired Student *t*‐test). (c, d) Hematoxylin–eosin (c) and Masson's trichrome (d) staining of aortic arch sections to quantify the number of nuclei in the media, the adventitia (adv.)/media thickness ratio, and collagen accumulation in the media (*n* = 5, two‐tailed unpaired Students *t*‐test). Bars, 250 μm. (e) Representative images of *en face* oil red O staining of aortas and quantification of atherosclerosis burden in aortic arch and thoracic aorta (*n* = 5, two‐tailed unpaired Student *t*‐test). Bar, 5 mm. (f) Representative images of Van Gieson staining of aortic valves and quantification of atherosclerosis burden measured as lesion area (μm^2^) and aortic perimeter affected by atherosclerosis (%) (*n* = 5, two‐tailed unpaired Student *t*‐test). Bar, 200 μm. HDL, high density lipoproteins; LDL, low density lipoproteins; L, lumen; PLT, platelets; RBC, red blood cells; WBC, white blood cells.

To test the effect of EC‐specific progerin expression on atherosclerosis development, we placed 2‐month‐old *Apoe* ^*−/−*^ *Lmna* ^*LCS/LCS*^ *Cdh5‐CreERT2* mice and *Apoe* ^*−/−*^ *Lmna* ^*LCS/LCS*^ controls on a high‐fat diet, and analyzed them at 4 months of age. Hematological parameters and serum lipid profiles were similar in both genotypes (Figure 6a, b, Figure S2), and histological analysis of aortic arch sections revealed no between‐genotype differences in medial VSMC and collagen content or adventitial thickness (Figure 6c, d). *En face* oil red O staining of aortas also showed a similar atherosclerosis burden in *Apoe* ^*−/−*^ *Lmna* ^*LCS/LCS*^ *Cdh5‐CreERT2* mice and *Apoe* ^*−/−*^ *Lmna* ^*LCS/LCS*^ controls (Figure 6e), and histological analysis of the aortic valves showed indistinguishable lesion area and percentage of perimeter affected by atherosclerosis in the two genotypes (Figure 6f).

## 4 DISCUSSION

Given the critical role played by ECs in cardiovascular pathophysiology, it is of interest to assess their contribution to HGPS‐associated cardiovascular disease in order to determine their suitability as therapeutic targets. Several groups have analyzed the role of progerin expression in ECs using a variety of in vitro systems. Overexpression of progerin, but not wild‐type lamin A, was found to increase proinflammatory gene expression, oxidative stress, DNA damage, and cellular senescence in human coronary artery ECs (Bidault et al., 2020). A separate study using the same experimental system also showed progerin‐induced DNA damage, but only in ECs with evident nuclear structural defects and not in progerin‐expressing ECs with apparently normal nuclei (Constantinescu et al., 2010). Human umbilical vein ECs overexpressing GFP‐tagged human progerin exhibited features of cellular senescence, nuclear abnormalities, higher expression of proinflammatory cytokines, and increased IL‐1β‐stimulated neutrophil adhesion compared with cells overexpressing GFP‐lamin A (Rolas et al., 2024). Moreover, the same progerin‐overexpressing human ECs were unable to adapt to shear stress, but this effect was also observed upon lamin A overexpression (Danielsson et al., 2022). Although informative, data obtained from overexpression systems can be difficult to interpret due to the presence of supraphysiological progerin levels. To circumvent this issue, investigators have used human stem cell‐derived ECs in which progerin expression is controlled by the endogenous *LMNA* promoter. Several studies have analyzed progerin‐expressing ECs differentiated from induced pluripotent stem cells generated from HGPS patient fibroblasts, comparing them with control ECs derived from healthy donors. In this system, progerin‐expressing ECs showed nuclear structural defects that were accompanied by elevated levels of reactive oxygen species, DNA damage, senescence, proinflammatory gene expression, and hypotonicity‐induced apoptosis, as well as depressed levels of proliferation, angiogenesis, lipid uptake, flow‐mediated gene expression, and nitric oxide production (Abutaleb et al., 2023; Atchison et al., 2020; Gete et al., 2021; Lo et al., 2014; Matrone et al., 2019; Mojiri et al., 2021; Xu et al., 2022). However, in other studies using the same system or ECs differentiated from human embryonic stem cells in which the HGPS‐causing mutation was introduced by gene editing, endothelial progerin expression did not alter proliferation, angiogenesis, lipid uptake, nitric oxide production, or genome stability (Wu et al., 2018; Zhang et al., 2011). Although progerin‐expressing ECs derived from human stem cells are a valuable experimental tool, they have several limitations. These include the non‐physiological context of in vitro assays, the potential for incomplete differentiation of stem cells into bona fide ECs, the failure to model the heterogeneity that exists between ECs from different tissues, and the use of a limited number of stem cell clones in each study. Understanding the effects of EC progerin expression in a complex multicellular context such as the cardiovascular system thus requires the use of in vivo systems.

Our mouse studies presented here show that progerin expression in ECs in vivo is not sufficient to induce features characteristic of HGPS such as defective postnatal growth, premature death, and cardiovascular alterations, including VSMC loss, an elevated adventitia/media ratio, medial collagen accumulation, excessive atherosclerosis, heart fibrosis, and cardiac dysfunction and electrical alterations. In addition, we previously reported that *Lmna* ^*LCS/LCS*^ *Tie2Cre* mice, which express progerin in ECs and some hematopoietic cell subsets, do not show increased vascular stiffness or impaired vascular tone compared with progerin‐free controls (Del Campo et al., 2019, 2020). However, the lack of effects of EC‐specific progerin expression in our models does not exclude the possibility that HGPS might alter EC function indirectly via cues from progerin‐expressing non‐endothelial cells. This possibility is supported by studies in *Lmna* ^*G609G/G609G*^ mice with ubiquitous progerin expression, which revealed defective endothelium‐dependent aortic relaxation in response to acetylcholine (Del Campo et al., 2020; Sun et al., 2020) and transcriptional changes in lung ECs related to the inflammatory response (Sun et al., 2020). Moreover, we recently demonstrated that aortic ECs in *Apoe* ^*−/−*^ *Lmna* ^*G609G/G609G*^ mice with ubiquitous progerin expression undergo endothelial‐to‐mesenchymal transition, a proatherogenic process that was also observed in *Apoe* ^*−/−*^ *Lmna* ^*LCS/LCS*^ *SM22αCre* mice with progerin expressed predominantly in VSMCs but not in *Apoe* ^*−/−*^ *Lmna* ^*LCS/LCS*^ *Cdh5‐CreERT2* mice with EC‐specific progerin expression (Hamczyk et al., 2024). Collectively, these findings suggest that progerin expression in ECs is not sufficient to induce cardiovascular anomalies and other HGPS‐associated alterations, and that progerin‐expressing dysfunctional VSMCs provoke EC changes that contribute to the HGPS‐associated cardiovascular phenotype.

Other mouse models of EC‐specific progerin expression have yielded some apparently conflicting results, both among them and with the data presented here. *Prog*‐*Tg* mice were designed to maintain endogenous lamin A and lamin C levels while expressing human progerin and lamin A in ECs under the control of a tetracycline‐responsive transcriptional activator regulated by the EC‐specific *Cdh5* promoter (Osmanagic‐Myers et al., 2019). These mice showed increased endothelial expression of pro‐fibrotic, proinflammatory, and senescence markers, an augmented inflammatory infiltrate in liver and lung, and altered bone microstructure (Fleischhacker et al., 2024; Manakanatas et al., 2022). In agreement with our previous studies in *Lmna* ^*LCS/LCS*^ *Tie2Cre* mice (Del Campo et al., 2020) and the results presented here from *Lmna* ^*LCS/LCS*^ *Cdh5‐CreERT2* and *Apoe* ^*−/−*^ *Lmna* ^*LCS/LCS*^ *Cdh5‐CreERT2* mice, EC‐specific progerin expression in *Prog‐Tg* mice did not induce defective acetylcholine‐mediated vasorelaxation or aortic VSMC loss. However, in sharp contrast with our data, *Prog‐Tg* mice showed reduced body weight, cardiac fibrosis, diastolic dysfunction, adventitial thickening, and premature death (Osmanagic‐Myers et al., 2019). In the *Lmna* ^*f/f*^;TC model, EC‐specific progerin expression was induced by Cre‐mediated recombination controlled by the *Tek* (a.k.a. *Tie2*) promoter (Sun et al., 2020), which is active in ECs and some hematopoietic cell types (Kisanuki et al., 2001; Payne et al., 2018). The specificity of EC progerin expression in *Lmna* ^*f*/*f*^;TC mice was not quantified, making it difficult to rule out progerin expression in non‐endothelial cell types in this model (Sun et al., 2020). Like *Prog‐Tg* mice, *Lmna* ^*f*/*f*^;TC mice showed body weight reduction and died prematurely. However, unlike *Lmna* ^*LCS/LCS*^ *Tie2Cre* and *Prog‐Tg* mice, *Lmna* ^*f*/*f*^;TC mice also exhibited reduced aortic vasorelaxation in response to acetylcholine (Sun et al., 2020). In another mouse model of *Tie2* promoter‐driven progerin expression (*Tie2‐Cre*;*Lmna* ^*LCS*/*LCS*^;*Rosa26* ^*tdTomato*/*tdTomato*^), ECs from cremasteric venules and the lung microvasculature showed elevated neutrophil attachment with a concomitant increase in vascular leakage, but these effects were only reported upon acute inflammation induced by IL‐1β or lipopolysaccharide. Indeed, in the absence of pro‐inflammatory stimuli, lungs from mice with EC‐specific progerin expression and progerin‐free controls showed no differences in the expression of a panel of 785 inflammation‐related genes (Rolas et al., 2024). This indicates that progerin presence in ECs by itself does not induce a major pro‐inflammatory endothelial state, in line with our findings showing no increased accumulation of leukocytes in the aortic intima from *Lmna* ^*LCS*/*LCS*^ *Cdh5‐CreERT2* mice (Figure 4c). Cardiac function was unaltered in *Lmna* ^*LCS*/*LCS*^ *Cdh5‐CreERT2* mice, whereas *Prog‐Tg* mice exhibited signs of diastolic dysfunction with preserved ejection fraction. Unlike both *Lmna* ^*LCS*/*LCS*^ *Cdh5*‐*CreERT2* and *Prog*‐*Tg* mice, *Lmna* ^*f*/*f*^;TC mice showed decreased heart rate and reduced ejection fraction. These discrepancies might reflect the different strategies used to induce EC‐specific progerin expression in each model, which could lead to model‐specific limitations. For example, *Tie2* promoter‐driven recombination in *Lmna* ^*LCS*/*LCS*^ *Tie2Cre* mice (Del Campo et al., 2019, 2020), *Lmna* ^*f*/*f*^;TC mice (Sun et al., 2020), and *Tie2*‐*Cre*;*Lmna* ^*LCS*/*LCS*^;*Rosa26* ^*tdTomato*/*tdTomato*^ mice (Rolas et al., 2024) is not fully specific to ECs and can occur in some leukocytes (Payne et al., 2018). Also, due to the genetic approach used to generate them, *Lmna* ^*f*/*f*^ and *Lmna* ^*LCS*/*LCS*^ mice maintained expression of lamin C and, unlike wild‐type mice, lacked lamin A (Osorio et al., 2011; Sun et al., 2020). Mice expressing lamin C while lacking lamin A were initially reported to be indistinguishable from wild‐type controls (Fong et al., 2006; Osorio et al., 2011), but they were later found to live longer and to have depressed energy metabolism, increased weight gain, and reduced respiration (Lopez‐Mejia et al., 2014). The absence of lamin A in *Lmna* ^*f*/*f*^ and *Lmna* ^*LC*S/*LCS*^ mice and their derivatives could therefore have as‐yet undefined effects. Despite this potential limitation, *Lmna* ^*LCS*/*LCS*^ *SM22αCre* and *Apoe* ^*−*/*−*^ *Lmna* ^*LCS*/*LCS*^ *SM22αCre* mice exhibit characteristics of HGPS, including adventitial thickening, medial VSMC loss, impaired vascular contraction, increased arterial stiffness, and exaggerated atherosclerosis (Del Campo et al., 2019, 2020; Hamczyk et al., 2019; Hamczyk et al., 2018). Thus, even with the possible limitations associated with *Lmna* ^*LCS*/*LCS*^ mice, progerin expression in VSMCs versus ECs with this system results in clearly different outcomes (the presence or lack of progeroid signs, respectively). A potential limitation of our strategy to induce EC‐specific progerin expression in 1.5‐month‐old mice is the lack of progerin in ECs during development and the first weeks after birth, which could alter the acquisition of the HGPS phenotype. However, whereas *Lmna* ^*G609G*/*G609G*^ mice with constitutive and ubiquitous progerin expression show increased arterial stiffness and altered vasoreactivity in response to phenylephrine or acetylcholine compared with progerin‐free controls, these alterations were not observed in age‐matched *Lmna* ^*LCS*/*LCS*^ *Tie2Cre* mice with constitutive progerin expression in ECs (Del Campo et al., 2019, 2020). Given the differences between mouse models of EC‐specific progerin expression, caution must be used when interpreting results derived from a single model. Our previous studies (Del Campo et al., 2019, 2020; Hamczyk et al., 2024) and the results presented here show that EC‐specific progerin expression did not alter any cardiovascular parameter tested in three mouse models (*Lmna* ^*LCS*/*LCS*^ *Tie2Cre*, *Lmna* ^*LCS*/*LCS*^ *Cdh5‐CreERT2*, and *Apoe* ^*−*/*−*^ *Lmna* ^*LCS*/*LCS*^ *Cdh5‐CreERT2*) that rely on two different strategies for EC‐specific Cre‐mediated recombination (*Tie2* or *Cdh5* promoter‐driven), either in atherosclerosis‐free or atheroprone mice (intact versus deleted *Apoe* gene). A clear advantage of *Lmna* ^*LCS*/*LCS*^‐derived models is that the endogenous mouse *Lmna* promoter drives progerin expression within the physiological range, whereas the expression of human progerin and lamin A in *Prog‐Tg* mice was approximately 4‐fold higher than that of endogenous mouse lamin A (Osmanagic‐Myers et al., 2019). We are therefore confident that our studies cast reasonable doubt on the previously reported causal role of EC‐specific progerin expression in the development of HGPS‐associated body weight reduction, premature death, and cardiovascular phenotypes, especially the development of atherosclerosis.

An alternative approach to testing the effects of progerin expression in specific cell types is to eliminate progerin in a cell–type‐specific manner in the context of ubiquitous progerin expression. To that end, we previously generated *HGPSrev* mice that ubiquitously express physiologically relevant amounts of progerin (expression driven by the endogenous mouse *Lmna* promoter), lack lamin A, and allow time‐ and cell–type‐specific progerin suppression and lamin A restoration upon Cre‐mediated recombination (Sanchez‐Lopez et al., 2021). Using this model, we showed that *SM22α* promoter‐driven progerin suppression and lamin A restoration in VSMCs prevents HGPS‐associated VSMC loss, vascular fibrosis, intimal leukocyte recruitment, aggravated atherosclerosis, and premature death (Benedicto et al., 2024; Sanchez‐Lopez et al., 2021). Conversely, *Cdh5*‐driven progerin suppression and lamin A restoration in ECs did not protect progeroid mice from any of the typical HGPS features (Benedicto et al., 2024). The results presented here, together with our previous results obtained in mouse models of VSMC‐ and EC‐specific progerin expression or suppression, strongly suggest that progerin expression in VSMCs, but not ECs, is a direct cause of HGPS cardiovascular pathology and premature death. These findings should be considered in the future design of gene‐editing therapies aimed at reducing progerin expression in HGPS patients (Beyret et al., 2019; Koblan et al., 2021; Santiago‐Fernandez et al., 2019; Whisenant et al., 2022), as these therapies may be safer and more efficient if targeted to specific cell types.

## AUTHOR CONTRIBUTIONS

I.B., M.R.H. and V.A. designed research; I.B., M.R.H, R.M.N., A.B., R.M.C., C.E.‐E., P.G., M.F.‐P., M.J.A‐M., and C.G.‐G performed research; I.B., M.R.H., and B.D. analyzed data; I.B. and V.A. wrote the paper; all authors revised the manuscript.

## FUNDING INFORMATION

Work supported by grant PID2022‐141211OB‐I00 funded by MICIU/AEI/10.13039/501100011033 and ERDF/EU to V.A. I.B. was supported by the Comunidad de Madrid (2017‐T1/BMD‐5247, 2021‐5A/BMD‐20944) with co‐funding from the European Structural and Investment Fund, RYC2021‐033805‐I (MICIU/AEI/10.13039/501100011033, European Union NextGenerationEU/PRTR), and PID2022‐137111OA‐I00 (MICIU/AEI/10.13039/501100011033, ERDF/EU). A.B. was supported by MICIU/AEI/10.13039/501100011033 and ESF (BES‐2017‐079705); C.E.‐E. by Fundación “la Caixa” (LCF/BQ/DR19/1170012), R.M.N by the Ministerio de Educación, Cultura y Deporte (FPU16/05027), and M.R.H. by the MICIU (IJC2019‐040798‐I), AIAS and Aarhus University Research Foundation. The CNIC is supported by Instituto de Salud Carlos III, the Ministerio de Ciencia, Innovación y Universidades (MICIU), and Pro‐CNIC Foundation, and is a Severo Ochoa Center of Excellence (grant CEX2020‐001041‐S funded by MICIU/AEI/10.13039/501100011033). Microscopy was conducted at the CNIC Microscopy Unit and ICTS (Unique Science and Technology Infrastructure)‐ReDiB supported by MICIU at TRIMA (MICIU/AEI/10.13039/501100011033 and the ERDF, A way to make Europe).

## CONFLICT OF INTEREST STATEMENT

All the contributing authors declared no conflicts of interest. The funders had no role in the design of the study, the collection, analysis, or interpretation of data, and reporting of the study.

## Supporting information


Figure S1.

Figure S2.

## Data Availability

All data supporting the findings of this study are available within the article.

## References

[acel14389-bib-0001] Abutaleb, N. O. , Atchison, L. , Choi, L. , Bedapudi, A. , Shores, K. , Gete, Y. , Cao, K. , & Truskey, G. A. (2023). Lonafarnib and everolimus reduce pathology in iPSC‐derived tissue engineered blood vessel model of Hutchinson‐Gilford progeria syndrome. Scientific Reports, 13(1), 5032. 10.1038/s41598-023-32035-3 36977745 PMC10050176

[acel14389-bib-0002] Atchison, L. , Abutaleb, N. O. , Snyder‐Mounts, E. , Gete, Y. , Ladha, A. , Ribar, T. , Cao, K. , & Truskey, G. A. (2020). iPSC‐derived endothelial cells affect vascular function in a tissue‐engineered blood vessel model of Hutchinson‐Gilford progeria syndrome. Stem Cell Reports, 14(2), 325–337. 10.1016/j.stemcr.2020.01.005 32032552 PMC7013250

[acel14389-bib-0003] Barettino, A. , Benedicto, I. , & Andres, V. (2022). Whole mount preparation of mouse aorta for confocal microscopy studies of the intima. Methods in Molecular Biology, 2419, 597–610. 10.1007/978-1-0716-1924-7_37 35237991

[acel14389-bib-0004] Benedicto, I. , Carmona, R. M. , Barettino, A. , Espinós‐Estévez, C. , Gonzalo, P. , Nevado, R. M. , de la Fuente‐Pérez, M. , Andrés‐Manzano, M. J. , González‐Gómez, C. , Rolas, L. , Dorado, B. , Nourshargh, S. , Hamczyk, M. R. , & Andrés, V. (2024). Exacerbated atherosclerosis in progeria is prevented by progerin elimination in vascular smooth muscle cells but not endothelial cells. Proceedings of the National Academy of Sciences, 121(18), e2400752121. 10.1073/pnas.2400752121 PMC11066978 38648484

[acel14389-bib-0005] Benedicto, I. , Dorado, B. , & Andres, V. (2021). Molecular and cellular mechanisms driving cardiovascular disease in Hutchinson‐Gilford progeria syndrome: Lessons learned from animal models. Cells, 10(5), 1157. 10.3390/cells10051157 PMC8151355 34064612

[acel14389-bib-0006] Beyret, E. , Liao, H. K. , Yamamoto, M. , Hernandez‐Benitez, R. , Fu, Y. , Erikson, G. , Reddy, P. , & Izpisua Belmonte, J. C. (2019). Single‐dose CRISPR‐Cas9 therapy extends lifespan of mice with Hutchinson‐Gilford progeria syndrome. Nature Medicine, 25(3), 419–422. 10.1038/s41591-019-0343-4 PMC6541418 30778240

[acel14389-bib-0007] Bidault, G. , Garcia, M. , Capeau, J. , Morichon, R. , Vigouroux, C. , & Bereziat, V. (2020). Progerin expression induces inflammation, oxidative stress and senescence in human coronary endothelial cells. Cells, 9(5), 1201. 10.3390/cells9051201 PMC7290406 32408587

[acel14389-bib-0008] Constantinescu, D. , Csoka, A. B. , Navara, C. S. , & Schatten, G. P. (2010). Defective DSB repair correlates with abnormal nuclear morphology and is improved with FTI treatment in Hutchinson‐Gilford progeria syndrome fibroblasts. Experimental Cell Research, 316(17), 2747–2759. 10.1016/j.yexcr.2010.05.015 20599958

[acel14389-bib-0009] Danielsson, B. E. , Peters, H. C. , Bathula, K. , Spear, L. M. , Noll, N. A. , Dahl, K. N. , & Conway, D. E. (2022). Progerin‐expressing endothelial cells are unable to adapt to shear stress. Biophysical Journal, 121(4), 620–628. 10.1016/j.bpj.2022.01.004 34999130 PMC8873939

[acel14389-bib-0010] De Sandre‐Giovannoli, A. , Bernard, R. , Cau, P. , Navarro, C. , Amiel, J. , Boccaccio, I. , Lyonnet, S. , Stewart, C. L. , Munnich, A. , Le Merrer, M. , & Levy, N. (2003). Lamin a truncation in Hutchinson‐Gilford progeria. Science, 300(5628), 2055. 10.1126/science.1084125 12702809

[acel14389-bib-0011] Del Campo, L. , Sanchez‐Lopez, A. , Gonzalez‐Gomez, C. , Andres‐Manzano, M. J. , Dorado, B. , & Andres, V. (2020). Vascular smooth muscle cell‐specific progerin expression provokes contractile impairment in a mouse model of Hutchinson‐Gilford progeria syndrome that is ameliorated by nitrite treatment. Cells, 9(3), 656. 10.3390/cells9030656 PMC7140649 32182706

[acel14389-bib-0012] Del Campo, L. , Sanchez‐Lopez, A. , Salaices, M. , von Kleeck, R. A. , Exposito, E. , Gonzalez‐Gomez, C. , Cusso, L. , Guzman‐Martinez, G. , Ruiz‐Cabello, J. , Desco, M. , Assoian, R. K. , Briones, A. M. , & Andres, V. (2019). Vascular smooth muscle cell‐specific progerin expression in a mouse model of Hutchinson‐Gilford progeria syndrome promotes arterial stiffness: Therapeutic effect of dietary nitrite. Aging Cell, 18(3), e12936. 10.1111/acel.12936 30884114 PMC6516150

[acel14389-bib-0013] Eriksson, M. , Brown, W. T. , Gordon, L. B. , Glynn, M. W. , Singer, J. , Scott, L. , Erdos, M. R. , Robbins, C. M. , Moses, T. Y. , Berglund, P. , Dutra, A. , Pak, E. , Durkin, S. , Csoka, A. B. , Boehnke, M. , Glover, T. W. , & Collins, F. S. (2003). Recurrent de novo point mutations in lamin A cause Hutchinson‐Gilford progeria syndrome. Nature, 423(6937), 293–298. 10.1038/nature01629 12714972 PMC10540076

[acel14389-bib-0014] Fanjul, V. , Jorge, I. , Camafeita, E. , Macias, A. , Gonzalez‐Gomez, C. , Barettino, A. , Dorado, B. , Andres‐Manzano, M. J. , Rivera‐Torres, J. , Vazquez, J. , Lopez‐Otin, C. , & Andres, V. (2020). Identification of common cardiometabolic alterations and deregulated pathways in mouse and pig models of aging. Aging Cell, 19(9), e13203. 10.1111/acel.13203 32729659 PMC7511870

[acel14389-bib-0015] Fleischhacker, V. , Milosic, F. , Bricelj, M. , Kührer, K. , Wahl‐Figlash, K. , Heimel, P. , Diendorfer, A. , Nardini, E. , Fischer, I. , Stangl, H. , Pietschmann, P. , Hackl, M. , Foisner, R. , Grillari, J. , Hengstschläger, M. , & Osmanagic‐Myers, S. (2024). Aged‐vascular niche hinders osteogenesis of mesenchymal stem cells through paracrine repression of Wnt‐axis. Aging Cell, *23*(6), e14139. 10.1111/acel.14139 38578073 PMC11166365

[acel14389-bib-0016] Fong, L. G. , Ng, J. K. , Lammerding, J. , Vickers, T. A. , Meta, M. , Cote, N. , Gavino, B. , Qiao, X. , Chang, S. Y. , Young, S. R. , Yang, S. H. , Stewart, C. L. , Lee, R. T. , Bennett, C. F. , Bergo, M. O. , & Young, S. G. (2006). Prelamin A and lamin A appear to be dispensable in the nuclear lamina. The Journal of Clinical Investigation, 116(3), 743–752. 10.1172/JCI27125 16511604 PMC1386109

[acel14389-bib-0017] Gete, Y. G. , Koblan, L. W. , Mao, X. , Trappio, M. , Mahadik, B. , Fisher, J. P. , Liu, D. R. , & Cao, K. (2021). Mechanisms of angiogenic incompetence in Hutchinson‐Gilford progeria via downregulation of endothelial NOS. Aging Cell, 20(7), e13388. 10.1111/acel.13388 34086398 PMC8282277

[acel14389-bib-0018] Gordon, L. B. , Brown, W. T. , & Collins, F. S. (1993). Hutchinson‐Gilford progeria syndrome. In M. P. Adam , J. Feldman, G. M. Mirzaa, R. A. Pagon , S. E. Wallace , & A. Amemiya (Eds.), GeneReviews^®^ . Seattle, WA: University of Washington. https://www.ncbi.nlm.nih.gov/pubmed/20301300 20301300

[acel14389-bib-0019] Hamczyk, M. R. , del Campo, L. , & Andres, V. (2018). Aging in the cardiovascular system: Lessons from Hutchinson‐Gilford progeria syndrome. Annual Review of Physiology, 80, 27–48. 10.1146/annurev-physiol-021317-121454 28934587

[acel14389-bib-0020] Hamczyk, M. R. , Nevado, R. M. , Gonzalo, P. , Andrés‐Manzano, M. J. , Nogales, P. , Quesada, V. , Rosado, A. , Torroja, C. , Sánchez‐Cabo, F. , Dopazo, A. , Bentzon, J. F. , López‐Otín, C. , & Andrés, V. (2024). Endothelial‐to‐mesenchymal transition contributes to accelerated atherosclerosis in Hutchinson‐Gilford progeria syndrome. Circulation. 10.1161/CIRCULATIONAHA.123.065768 39206565

[acel14389-bib-0021] Hamczyk, M. R. , Villa‐Bellosta, R. , Gonzalo, P. , Andres‐Manzano, M. J. , Nogales, P. , Bentzon, J. F. , Lopez‐Otin, C. , & Andres, V. (2018). Vascular smooth muscle‐specific progerin expression accelerates atherosclerosis and death in a mouse model of Hutchinson‐Gilford progeria syndrome. Circulation, 138(3), 266–282. 10.1161/CIRCULATIONAHA.117.030856 29490993 PMC6075893

[acel14389-bib-0022] Hamczyk, M. R. , Villa‐Bellosta, R. , Quesada, V. , Gonzalo, P. , Vidak, S. , Nevado, R. M. , Andres‐Manzano, M. J. , Misteli, T. , Lopez‐Otin, C. , & Andres, V. (2019). Progerin accelerates atherosclerosis by inducing endoplasmic reticulum stress in vascular smooth muscle cells. EMBO Molecular Medicine, 11(4), e9736. 10.15252/emmm.201809736 PMC6460349 30862662

[acel14389-bib-0023] Kisanuki, Y. Y. , Hammer, R. E. , Miyazaki, J. , Williams, S. C. , Richardson, J. A. , & Yanagisawa, M. (2001). Tie2‐Cre transgenic mice: A new model for endothelial cell‐lineage analysis in vivo. Developmental Biology, 230(2), 230–242. 10.1006/dbio.2000.0106 11161575

[acel14389-bib-0024] Koblan, L. W. , Erdos, M. R. , Wilson, C. , Cabral, W. A. , Levy, J. M. , Xiong, Z.‐M. , Tavarez, U. L. , Davison, L. M. , Gete, Y. G. , Mao, X. , Newby, G. A. , Doherty, S. P. , Narisu, N. , Sheng, Q. , Krilow, C. , Lin, C. Y. , Gordon, L. B. , Cao, K. , Collins, F. S. , … Liu, D. R. (2021). In vivo base editing rescues Hutchinson‐Gilford progeria syndrome in mice. Nature, 589(7843), 608–614. 10.1038/s41586-020-03086-7 33408413 PMC7872200

[acel14389-bib-0025] Lo, C.‐Y. , Tjong, Y.‐W. , Ho, J. C.‐Y. , Siu, C.‐W. , Cheung, S.‐Y. , Tang, N. L. , Yu, S. , Tse, H.‐F. , & Yao, X. (2014). An upregulation in the expression of vanilloid transient potential channels 2 enhances hypotonicity‐induced cytosolic Ca^2+^ rise in human induced pluripotent stem cell model of Hutchinson‐Gillford progeria. PLoS One, 9(1), e87273. 10.1371/journal.pone.0087273 24475260 PMC3903625

[acel14389-bib-0026] Lopez‐Mejia, I. C. , de Toledo, M. , Chavey, C. , Lapasset, L. , Cavelier, P. , Lopez‐Herrera, C. , Chebli, K. , Fort, P. , Beranger, G. , Fajas, L. , Amri, E. Z. , Casas, F. , & Tazi, J. (2014). Antagonistic functions of LMNA isoforms in energy expenditure and lifespan. EMBO Reports, 15(5), 529–539. 10.1002/embr.201338126 24639560 PMC4210101

[acel14389-bib-0027] Manakanatas, C. , Ghadge, S. K. , Agic, A. , Sarigol, F. , Fichtinger, P. , Fischer, I. , Foisner, R. , & Osmanagic‐Myers, S. (2022). Endothelial and systemic upregulation of miR‐34a‐5p fine‐tunes senescence in progeria. Aging, 14(1), 195–224. 10.18632/aging.203820 35020601 PMC8791216

[acel14389-bib-0028] Matrone, G. , Thandavarayan, R. A. , Walther, B. K. , Meng, S. , Mojiri, A. , & Cooke, J. P. (2019). Dysfunction of iPSC‐derived endothelial cells in human Hutchinson‐Gilford progeria syndrome. Cell Cycle, 18(19), 2495–2508. 10.1080/15384101.2019.1651587 31411525 PMC6738911

[acel14389-bib-0029] McClintock, D. , Gordon, L. B. , & Djabali, K. (2006). Hutchinson‐Gilford progeria mutant lamin A primarily targets human vascular cells as detected by an anti‐lamin A G608G antibody. Proceedings of the National Academy of Sciences of the United States of America, 103(7), 2154–2159. 10.1073/pnas.0511133103 16461887 PMC1413759

[acel14389-bib-0030] Mojiri, A. , Walther, B. K. , Jiang, C. , Matrone, G. , Holgate, R. , Xu, Q. , Morales, E. , Wang, G. , Gu, J. , Wang, R. , & Cooke, J. P. (2021). Telomerase therapy reverses vascular senescence and extends lifespan in progeria mice. European Heart Journal, 42(42), 4352–4369. 10.1093/eurheartj/ehab547 34389865 PMC8603239

[acel14389-bib-0031] Olive, M. , Harten, I. , Mitchell, R. , Beers, J. K. , Djabali, K. , Cao, K. , Erdos, M. R. , Blair, C. , Funke, B. , Smoot, L. , Gerhard‐Herman, M. , Machan, J. T. , Kutys, R. , Virmani, R. , Collins, F. S. , Wight, T. N. , Nabel, E. G. , & Gordon, L. B. (2010). Cardiovascular pathology in Hutchinson‐Gilford progeria: Correlation with the vascular pathology of aging. Arteriosclerosis, Thrombosis, and Vascular Biology, 30(11), 2301–2309. 10.1161/ATVBAHA.110.209460 20798379 PMC2965471

[acel14389-bib-0032] Osmanagic‐Myers, S. , Kiss, A. , Manakanatas, C. , Hamza, O. , Sedlmayer, F. , Szabo, P. L. , Fischer, I. , Fichtinger, P. , Podesser, B. K. , Eriksson, M. , & Foisner, R. (2019). Endothelial progerin expression causes cardiovascular pathology through an impaired mechanoresponse. The Journal of Clinical Investigation, 129(2), 531–545. 10.1172/JCI121297 30422822 PMC6355303

[acel14389-bib-0033] Osorio, F. G. , Navarro, C. L. , Cadinanos, J. , Lopez‐Mejia, I. C. , Quiros, P. M. , Bartoli, C. , Rivera, J. , Tazi, J. , Guzman, G. , Varela, I. , Depetris, D. , de Carlos, F. , Cobo, J. , Andres, V. , De Sandre‐Giovannoli, A. , Freije, J. M. , Levy, N. , & Lopez‐Otin, C. (2011). Splicing‐directed therapy in a new mouse model of human accelerated aging. Science Translational Medicine, 3(106), 106ra107. 10.1126/scitranslmed.3002847 22030750

[acel14389-bib-0034] Payne, S. , De Val, S. , & Neal, A. (2018). Endothelial‐specific Cre mouse models. Arteriosclerosis, Thrombosis, and Vascular Biology, 38(11), 2550–2561. 10.1161/ATVBAHA.118.309669 30354251 PMC6218004

[acel14389-bib-0035] Rolas, L. , Stein, M. , Barkaway, A. , Reglero‐Real, N. , Sciacca, E. , Yaseen, M. , Wang, H. , Vazquez‐Martinez, L. , Golding, M. , Blacksell, I. A. , Giblin, M. J. , Jaworska, E. , Bishop, C. L. , Voisin, M.‐B. , Gaston‐Massuet, C. , Fossati‐Jimack, L. , Pitzalis, C. , Cooper, D. , Nightingale, T. D. , … Nourshargh, S. (2024). Senescent endothelial cells promote pathogenic neutrophil trafficking in inflamed tissues. EMBO Reports, 25, 3842–3869. 10.1038/s44319-024-00182-x 38918502 PMC11387759

[acel14389-bib-0036] Sanchez‐Lopez, A. , Espinos‐Estevez, C. , Gonzalez‐Gomez, C. , Gonzalo, P. , Andres‐Manzano, M. J. , Fanjul, V. , Riquelme‐Borja, R. , Hamczyk, M. R. , Macias, A. , Del Campo, L. , Camafeita, E. , Vazquez, J. , Barkaway, A. , Rolas, L. , Nourshargh, S. , Dorado, B. , Benedicto, I. , & Andres, V. (2021). Cardiovascular progerin suppression and lamin A restoration rescue Hutchinson‐Gilford progeria syndrome. Circulation, 144(22), 1777–1794. 10.1161/CIRCULATIONAHA.121.055313 34694158 PMC8614561

[acel14389-bib-0037] Santiago‐Fernandez, O. , Osorio, F. G. , Quesada, V. , Rodriguez, F. , Basso, S. , Maeso, D. , Rolas, L. , Barkaway, A. , Nourshargh, S. , Folgueras, A. R. , Freije, J. M. P. , & Lopez‐Otin, C. (2019). Development of a CRISPR/Cas9‐based therapy for Hutchinson‐Gilford progeria syndrome. Nature Medicine, 25(3), 423–426. 10.1038/s41591-018-0338-6 PMC6546610 30778239

[acel14389-bib-0038] Schindelin, J. , Arganda‐Carreras, I. , Frise, E. , Kaynig, V. , Longair, M. , Pietzsch, T. , Preibisch, S. , Rueden, C. , Saalfeld, S. , Schmid, B. , Tinevez, J.‐Y. , White, D. J. , Hartenstein, V. , Eliceiri, K. , Tomancak, P. , & Cardona, A. (2012). Fiji: An open‐source platform for biological‐image analysis. Nature Methods, 9(7), 676–682. 10.1038/nmeth.2019 22743772 PMC3855844

[acel14389-bib-0039] Sörensen, I. , Adams, R. H. , & Gossler, A. (2009). DLL1‐mediated Notch activation regulates endothelial identity in mouse fetal arteries. Blood, 113(22), 5680–5688. 10.1182/blood-2008-08-174508 19144989

[acel14389-bib-0040] Sun, S. , Qin, W. , Tang, X. , Meng, Y. , Hu, W. , Zhang, S. , Qian, M. , Liu, Z. , Cao, X. , Pang, Q. , Zhao, B. , Wang, Z. , Zhou, Z. , & Liu, B. (2020). Vascular endothelium‐targeted Sirt7 gene therapy rejuvenates blood vessels and extends life span in a Hutchinson‐Gilford progeria model. Science Advances, 6(8), eaay5556. 10.1126/sciadv.aay5556 32128409 PMC7030934

[acel14389-bib-0041] Whisenant, D. , Lim, K. , Revêchon, G. , Yao, H. , Bergo, M. O. , Machtel, P. , Kim, J.‐S. , & Eriksson, M. (2022). Transient expression of an adenine base editor corrects the Hutchinson‐Gilford progeria syndrome mutation and improves the skin phenotype in mice. Nature Communications, 13(1), 3068. 10.1038/s41467-022-30800-y PMC9163128 35654881

[acel14389-bib-0042] Wu, Z. , Zhang, W. , Song, M. , Wang, W. , Wei, G. , Li, W. , Lei, J. , Huang, Y. , Sang, Y. , Chan, P. , Chen, C. , Qu, J. , Suzuki, K. , Belmonte, J. C. I. , & Liu, G. H. (2018). Differential stem cell aging kinetics in Hutchinson‐Gilford progeria syndrome and Werner syndrome. Protein & Cell, 9(4), 333–350. 10.1007/s13238-018-0517-8 29476423 PMC5876188

[acel14389-bib-0043] Xu, Q. , Mojiri, A. , Boulahouache, L. , Morales, E. , Walther, B. K. , & Cooke, J. P. (2022). Vascular senescence in progeria: Role of endothelial dysfunction. European Heart Journal Open, 2(4), oeac047. 10.1093/ehjopen/oeac047 36117952 PMC9472787

[acel14389-bib-0044] Xu, S. , Ilyas, I. , Little, P. J. , Li, H. , Kamato, D. , Zheng, X. , Luo, S. , Li, Z. , Liu, P. , Han, J. , Harding, I. C. , Ebong, E. E. , Cameron, S. J. , Stewart, A. G. , & Weng, J. (2021). Endothelial dysfunction in atherosclerotic cardiovascular diseases and beyond: From mechanism to pharmacotherapies. Pharmacological Reviews, 73(3), 924–967. 10.1124/pharmrev.120.000096 34088867

[acel14389-bib-0045] Zhang, J. , Lian, Q. , Zhu, G. , Zhou, F. , Sui, L. , Tan, C. , Mutalif, R. A. , Navasankari, R. , Zhang, Y. , Tse, H. F. , Stewart, C. L. , & Colman, A. (2011). A human iPSC model of Hutchinson Gilford progeria reveals vascular smooth muscle and mesenchymal stem cell defects. Cell Stem Cell, 8(1), 31–45. 10.1016/j.stem.2010.12.002 21185252

