# Supplementary figures and images for: Endothelial cell‐specific progerin expression does not cause cardiovascular alterations and premature death
Source: Aging Cell. 2024 Oct 31;24(2):e14389. doi: 10.1111/acel.14389 (PMC11822624; doi:10.1111/acel.14389)

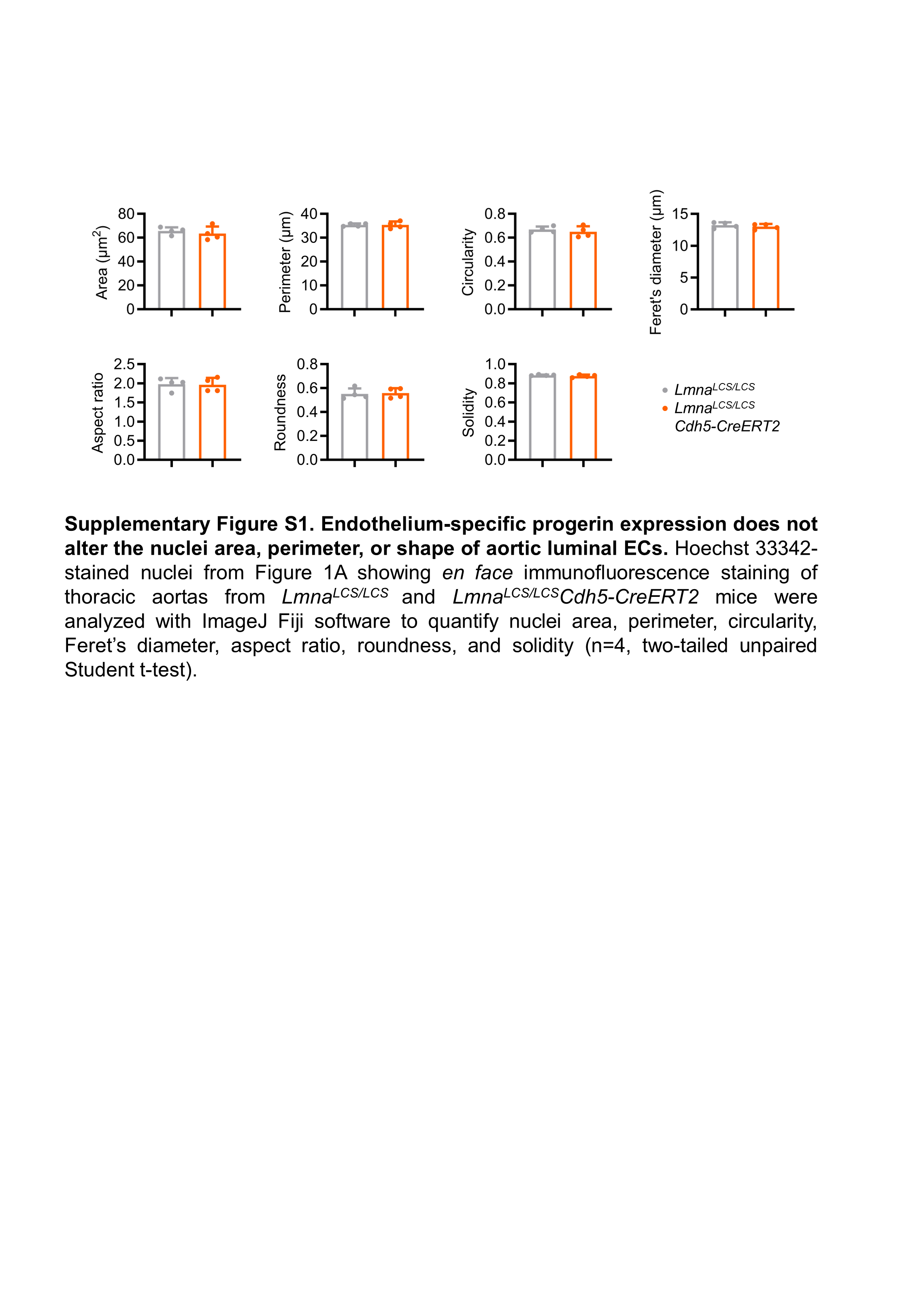

Supplement: Supplementary file 1 — Figure S1. [file ACEL-24-e14389-s002.tif]

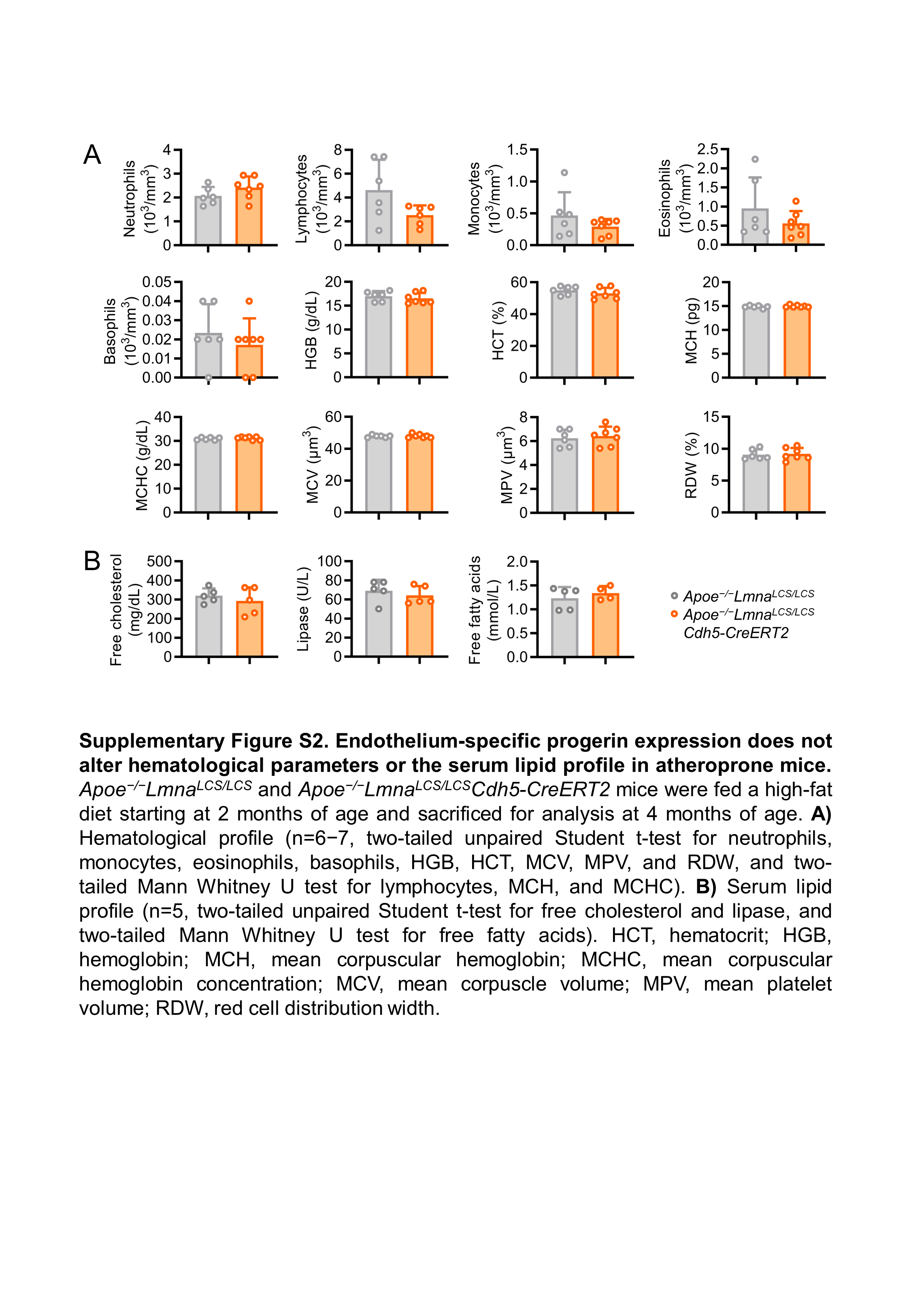

Supplement: Supplementary file 2 — Figure S2. [file ACEL-24-e14389-s001.tif]
